# Association between smoking and non-alcoholic fatty liver disease in Southeast Asia

**DOI:** 10.3389/fpubh.2022.1008878

**Published:** 2022-12-13

**Authors:** Hassan Mumtaz, Madiha Hameed, Abdul Basit Sangah, Amraha Zubair, Mohammad Hasan

**Affiliations:** ^1^Health Services Academy, Islamabad, Pakistan; ^2^Department of Medicine, AJK Medical College, Muzaffarabad, Pakistan; ^3^Department of Medicine, Liaquat National Medical College, Karachi, Pakistan; ^4^Department of Medicine, Dow University of Health Sciences, Karachi, Pakistan; ^5^Department of Medicine, Jinnah Post Graduate Medical Centre, Karachi, Pakistan

**Keywords:** nonalcoholic fatty liver disease, smoking, hepatocellular carcinoma, electronic cigarette, depression, cardiovascular disease, Southeast Asia

## Abstract

An estimated 8 million people die each year from tobacco smoking, with an increasing frequency recently being observed in Southeast Asian countries, which is a preventable risk factor for mortality. NAFLD, fibrosis, advancement of hepatocellular carcinoma, and prognosis for those with severe liver disease are all negatively influenced. NAFLD and cigarette usage seem to be a direct link. Oxidative stress and oncogenic signals have been implicated in cancer development in animal models and human clinical trials. The elevated risk of cardiovascular disease and malignancies in those with steatohepatitis and those who have had liver transplants is exacerbated by smoking. We found that smoking cessation may increase treatment response and fibrosis regression rates, decrease hepatocellular carcinoma incidence, and improve liver transplant outcomes. In the last segment, we'll look at electronic cigarettes, a hot subject in public health right now, as well as additional repercussions of smoking.

## Introduction

Although non-alcoholic fatty liver disease (NAFLD) was prevalent for decades in westerners, recent studies have shown the growing incidence of NAFLD in Southeast Asia, from 9% ranging to 45%, particularly in Pakistan, Sri Lanka, India, Nepal, Bangladesh, with the second largest proportion of cancer death attributable to HCC in East Asia (1, 2). South Korea also showed a rising frequency between 2006 (18.7%) and 2010 (27.3%) (3). Furthermore, a whopping 33.9% increase in NAFLD prevalence was reported between 2012 to 2017 in Southeast Asia (3). NAFLD affects an estimated 20–30% of Westerners (4, 5). NAFLD may lead to cirrhosis of the liver and hepatocellular cancer if it is not addressed. The name “NAFLD” encompasses all of these conditions (4–9). Type 2 diabetes mellitus, as well as waist circumferences >102 cm for men and 88 cm for women, have been linked to obesity (4–7, 9). Obese people have a 4.6-fold increase in the prevalence of NAFLD (7). NAFLD is linked not just to metabolic abnormalities but also bad lifestyle choices (5). Also, population aging accelerates the progression of NAFLD (10, 11). Thus as evidenced by the abdominal age predictor, AbdAge model, which was developed on liver MRI images and revealed that with advanced age, the liver becomes darker, its volume diminishes, and blood flow declines (10). Smoking and, more recently, NAFLD have been identified as risk factors for reflux esophagitis (12). NAFLD is presently a significant financial burden on the global healthcare system.

**Figure 1:**
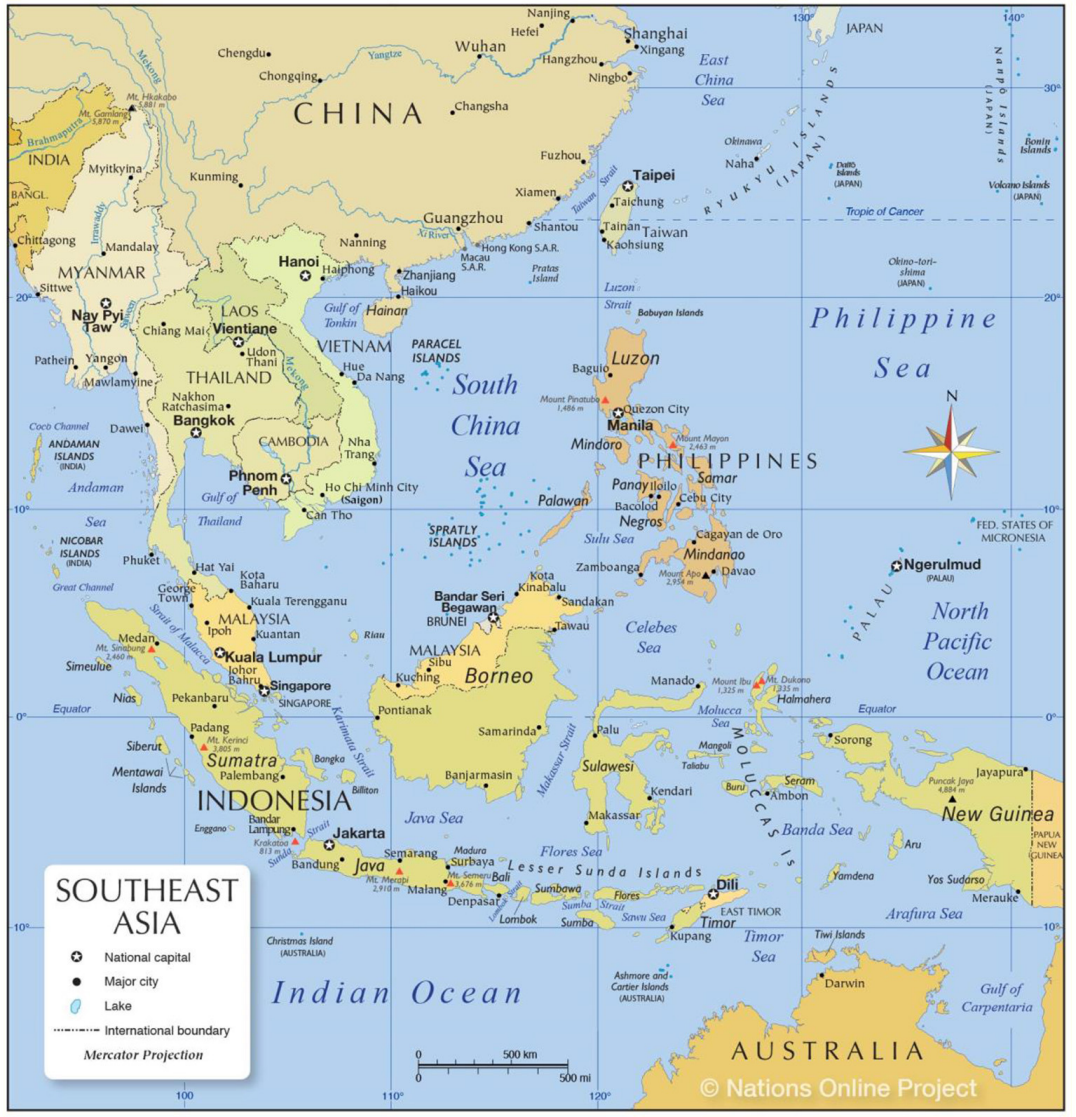
A map of Southeast Asia (35).

The burden of tobacco abuse in the region of Southeast Asia has remarkably soared in recent years, with approximately 400 million users, and resulted in about 1.2 million deaths per year (13–17). Cigarettes contain nearly 4,000 hazardous compounds, many of which are liver-damaging and habit-forming (4). Chronic liver disease, such as alcoholic liver disease, primary biliary cirrhosis, hepatitis B and C, and other chronic liver diseases, may be accelerated by tobacco use in addition to cardiovascular disease, type 2 diabetes, and hepatocellular cancer. (6, 7, 11). It's still unclear how smoking contributes to nonalcoholic fatty liver disease (NAFLD). In this article, the pathophysiology of smoking and non-alcoholic fatty liver disease (NAFLD) is discussed, and evidence is drawn from related studies, with special emphasis on data related to Southeast Asia. A cause-and-effect relationship between smoking and NAFLD could be best explained by the negative effects of potential confounders. A map of Southeast Asia has been shown in Figure 1.

## Smoking and NAFLD

**Figure 2:**
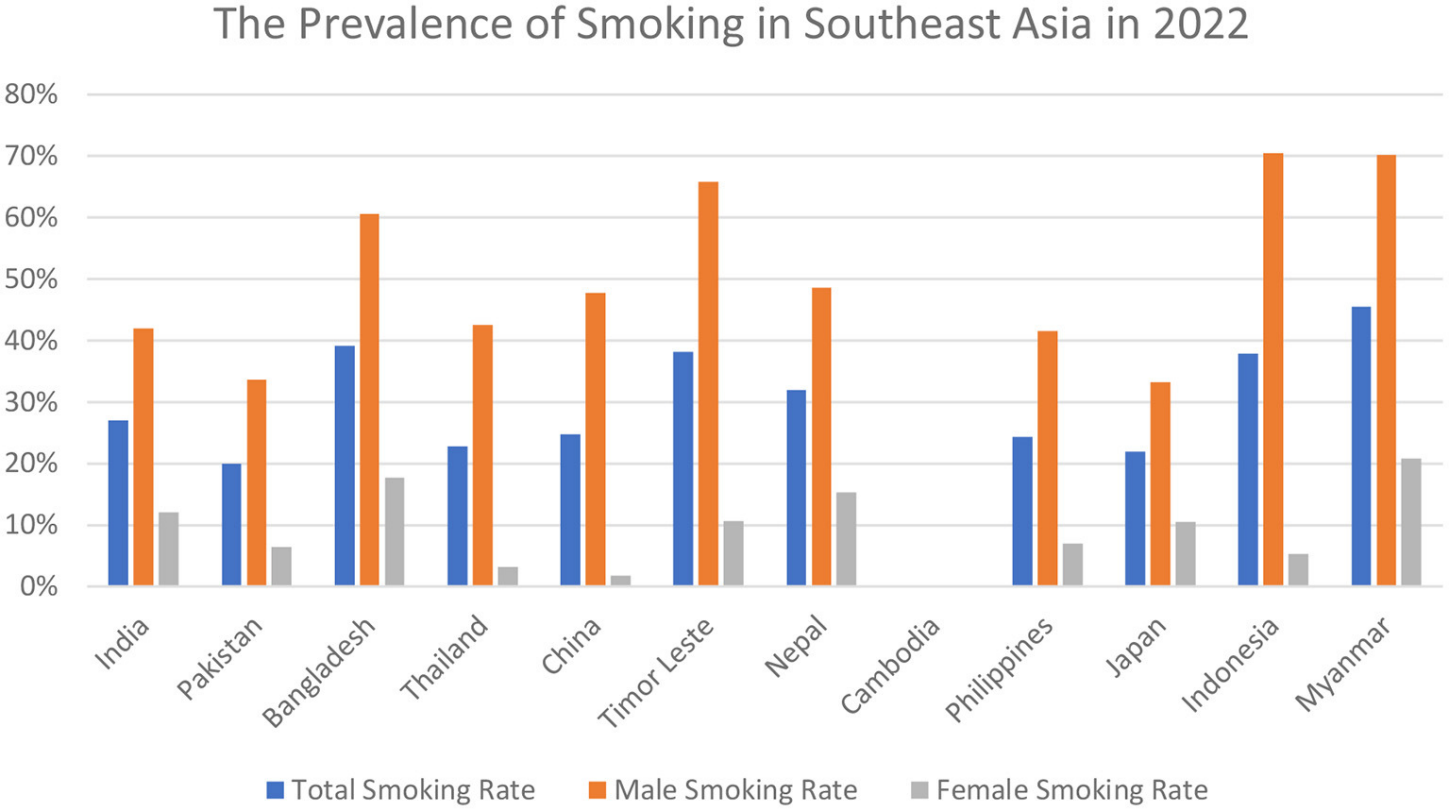
The latest 2022 prevalence rates of total smoking; male smoking; and female smoking in Southeast Asia.

Kishore et al. evaluated the frequency of hardcore smoking among three Southeast Asian countries, with India reporting the highest number of hardcore smokers as compared to Bangladesh, and Thailand, among a total of 31.3 million individuals (14). A serious public health issue, smoking kills more than 8 million people every year, and is a preventable cause of early disability and morbid mortality (15, 16). According to a 2017 research, the percentage of men and women who smoke every day is 25.0 percent and 5.4 percent, respectively. In 2015, smoking was directly responsible for the deaths of 6.4 million people, or 11.5% of the population (7). In the recent decade, several nations have seen an increase in the number of people who smoke (7). 36 percent of malignancies and 21 percent of all-cause fatalities in China have been linked to smoking cigarettes (11). Half of all males over the age of 18 in Europe smoke, ranging from 63% in Russia to 17% in Sweden (7). In developed countries, 24 percent of women smoke, compared to 7% in poor ones (7). Moreover, as the number of pack-years increased, the risk association also increased (10–19.9 pack-years: hazard ratio [HR] 1.25; 95% CI 1.21–1.29; >–20 pack-years: HR 1.36; 95% CI 1.30–1.42, compared to 0 pack-years) (4). According to demographics and health surveys, considering the Southeast Asian regions, among men, Indonesia ranked the highest smoking country (72.3%), whereas the percentages in Timor Leste, Bangladesh, and Maldives were 69.5, 60.0, and 47.3%, respectively (13). However, India, Pakistan, Cambodia, and Nepal reported the decreased trend with percentages as follows: 34.1, 31.6, 34.7, and 33.6% accordingly (13). While women smoked less frequently in every country than men, the studies reported Nepal the highest (9.8%), with Maldives (4.6%), Philippines (5.2%) and Pakistan (4.02%) following the trend (13). The latest epidemiologic data regarding the prevalence rates of smoking in Southeast Asia is shown in Figure 2 (17).

**Figure 3:**
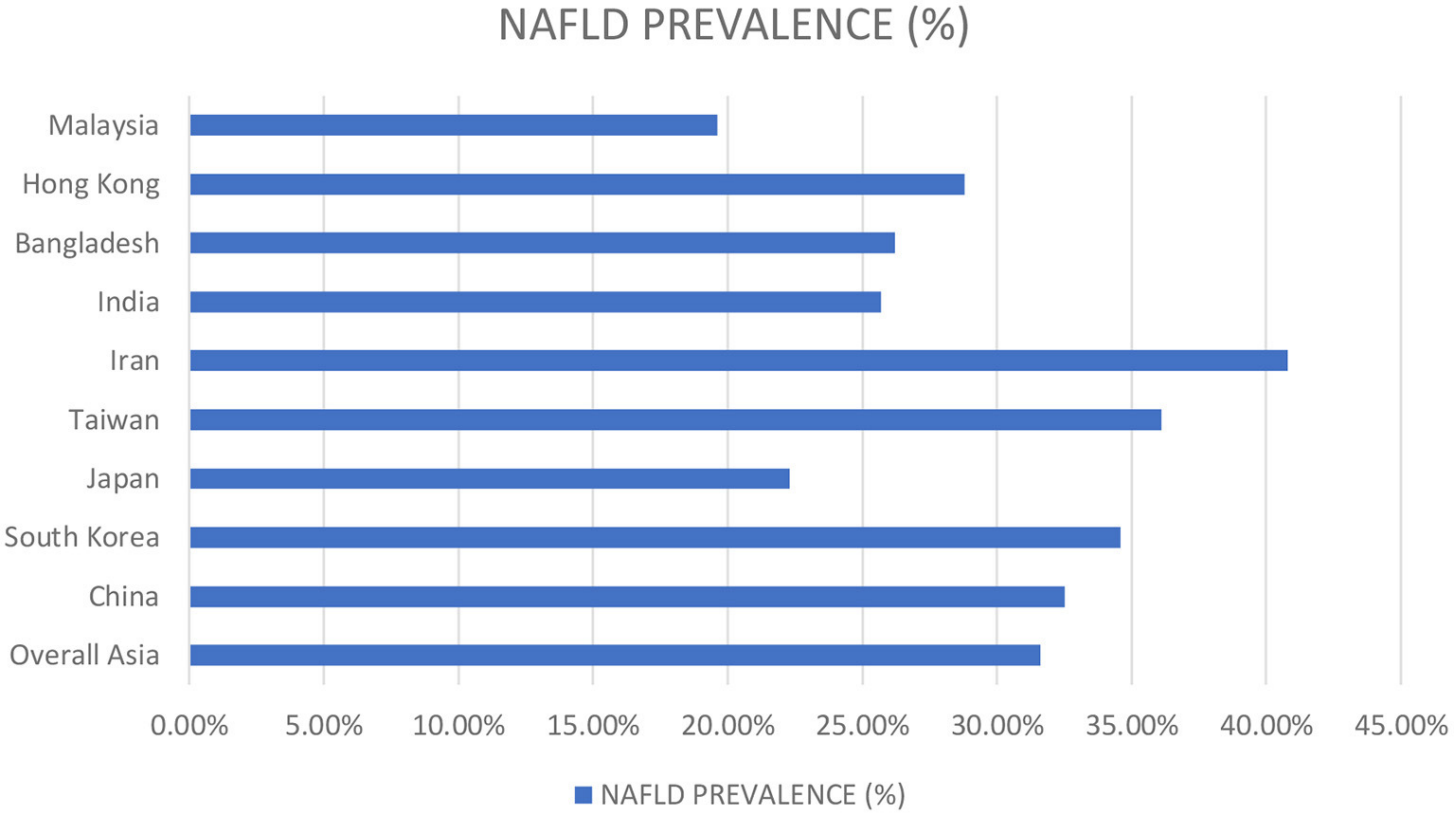
Demonstrates the prevalence of NAFLD in Asia.

Several studies have reported the high NAFLD predominance in males. Around 17.7% of females and 41% of males reported NAFLD in a study done in Japan. Similarly, Williams et al. reported a NAFLD prevalence of 58.9% among males in a prospective study conducted in the United States (18). Nevertheless, the males demonstrated high prevalence rates of NAFLD at all ages than females, but increasing age strikingly raised the frequency among females, with 3.3% in the twenties to 31.3% beyond the sixties. Data from China demonstrated parallel trends (19). This could possibly be explained by a greater increase in AST/ALT ratio in women relative to NAFLD-diagnosed men, predisposing women to an enhanced inflammatory response as the disease progresses and an increased risk for advanced fibrosis (20). Furthermore, endocrine factors play a role in determining the propensity to develop NAFLD in males, whereas, in females, estrogen is protective during the fertile period of life while the risk raises exponentially in post-menopausal women (21). The prevalence rates of NAFLD concerning the vast region of Asia are summarized in Figure 3 (22).

The pathogenic processes associated with smoking are recognized at the cellular, histologic, systemic, and clinical levels, respectively, increasing hepatocarcinogenesis, hepatic fibrosis, metabolic fatty liver disorders, and negatively affecting liver-related outcomes (7, 16). Numerous studies show a link between smoking and cirrhosis, chronic hepatitis B (CHB) infection, however, the connection between smoking and NAFLD is still debatable (5). Southeast Asian regions are significantly reporting the risk of smoking as a leading cause of developing NAFLD. A cohort study conducted in Japan among non-alcoholics demonstrated that cigarette smoking is strongly linked with the development of NAFLD, insulin resistance being the culprit (23). Similarly, Liu et al. observed in a 40-year-old Chinese population that heavy active, as well as passive cigarette smoking, correlated with NAFLD (24). The result from several studies conducted in China, Japan, and South Korea for over a decade indicated that a dose exposure risk existed for hepatic disease and smoking even when confounding factors were adjusted (25). Smoking has been linked to NAFLD by research conducted in Kagoshima, Japan by Hamabe et al. (26). Researchers have found a strong link between smoking and increased risk of NAFLD-related hepatic fibrosis in a Chinese study published in JAMA Hepatology by Hongjie Ou et al. (5). Smoking is strongly linked to NAFLD, according to a meta-analysis. Because stopping smoking increases weight and BMI, those who were smokers before are more likely to develop NAFLD (7). Passive smoking, on the other hand, raises the incidence of NAFLD by 1.38-fold because sidestream smoke includes more dangerous compounds than mainstream smoke (7). As a consequence of cigarette smoke's counterproductive estrogenic effects, which alter body fat composition, smoking-induced NAFLD may have an independent influence on BMI concerning NAFLD (7). On the basis of self-reports, pack-years, and urinary cotinine levels, a large South Korean cohort study conducted at Kangbuk Samsung Hospital concluded that current smoking was significantly and independently associated with an increased risk of NAFLD and fibrosis in healthy young and middle-aged men and women (6). Another Korean study by Kim et al. found a connection between NAFLD and urine cotinine confirmed smoking (4). NAFLD-affected women showed that current smoking led to a dramatic rise in NAFLD-related deaths as demonstrated by a cohort conducted among Thai civilians (27).

Numerous factors, some of which are yet understood, might relate smoking to NAFLD. Cytotoxic chemicals may accelerate the growth and proliferation of fibroblasts, which results in the formation of scar tissue (6). Moreover, nicotine strongly stimulates hepatic injury and subsequent fibrogenesis, *via* activation of nicotinic acetylcholine receptors (nAChR) (28). Hepatic stellate cells (HSCs) normally remain in a non-proliferative and inactive state, until injured by a harmful moiety. During the phase of nicotine-derived hepatocyte injury, transforming growth factor-β (TGF-β) is activated from multiple sources, including extracellular matrix (ECM), platelets, and macrophages. Consequently, activating and differentiating HSCs into myofibroblasts, which produce excessive fibrillar ECM proteins, leading to collagen I and III depositions in the space of Disse, thereby enhancing rapid fibrosis progression. Therefore, liver sinusoidal endothelial cells (LSEC), in a process called LSEC capillarization, defenestrate and form a basement membrane, subsequently predisposing to defective nutrient transport between hepatocytes and sinusoidal blood i.e., physiological disturbance (29). Likewise, Soeda and colleagues concluded that the use of the nAChR antagonist has shown promising outcomes in reversing the nicotine-mediated TGF-β production, therefore confirming the potential effect of TGF-β-induced fibrogenesis (30). Moreover, in response to microvasculature perturbations, like endothelial injury and subsequent hepatic tissue hypoxia, collagen-type I and vascular endothelial growth factor increase, thereby fibrosis ensues (16). Cigarette smoke also causes bacterial translocation following intestinal dysbiosis, and thus activates the Toll-like receptor 4 on the HSCs, enhancing fibrogenesis. Additionally, nicotine adversely affects the humoral and cell-mediated immune responses, leading to suppressed antibody formation caused by lymphocyte apoptosis. Moreover, smoking decreases CD4+ cell, and increases CD8+ cytotoxic T cells, which could be reversed by smoking cessation (31). Smokers' NAFLD is exacerbated by elevated levels of proinflammatory cytokines such as interleukin-1, IL-6, and tumor necrosis factor (6). Increased levels of FFAs in the circulation are a result of alterations in fat metabolism that lead to extraordinarily high rates of lipolysis (11). In the liver and adipose tissue, eight Triglycerides are formed when FFAs are metabolized by fat cells insulin resistance (IR) (7). Fat buildup, skeletal muscle FFA generation, impaired glucose absorption, and delayed gluconeogenesis all contribute to IR in NAFLD patients (7). Even if you lose weight, smoking may raise your chance of developing central obesity because it affects the distribution of fat in your body (6). In addition to reduced lipoprotein lipase activity, higher 3-hydroxy-3-methylglutaryl CoA reductase activity, and a decreased glucose-6-phosphatase dehydrogenase activity all contribute to dyslipidemia (11). Therefore, smoking leads to NAFLD development by increasing the bloodstream production of insulin-antagonistic hormones such as catecholamine and glucagon, which may lead to insulin resistance (6, 11). The development of liver disease caused by a high-fat diet may be worsened as a result of this effect, which may include mechanisms such as an increase in oxidative stress and cell death in the liver, as well as a decrease in the activation of AMPK, fatty acid synthase (*de novo* liver steatosis), and the 1-c sterol response element binding protein, all of which are linked to the development of liver disease (6, 16).

The trend of NAFLD in India is especially attributed to various factors like central obesity, higher BMI, hypertension, hypercholesterolemia, hypertriglyceridemia, hyperinsulinemia, and diabetes, as reported by Nigam et al. (1). The urban society in Sri Lanka showed a 32.6% prevalence of NAFLD with the same risk factors playing the role as in India, in addition to, insulin resistance, transaminitis and acanthosis nigricans (1). Central obesity proved to be the biggest threat among all the factors associated with NAFLD in Bangladesh, where 92.6% NAFLD affected were females (1). A study conducted in Nepal by Mittal et al. conveyed that 17% of the population had NAFLD; attributable to increased concentrations of serum alanine aminotransferase and aspartate aminotransferase (1). Economic growth and urbanization have led to an increased prevalence of NAFLD in Pakistan, approximately 42% in upper societies, along with promoting factors such as type 2 diabetes, hypertriglyceridemia, transaminitis, hepatomegaly, and high BMI (1). In a study conducted in Peshawar, Pakistan, around 47% patients reported NAFLD, while other different studies reported 14% prevalence (32). In a cohort study in Japan, Liu reported that approximately 40% of females and 25% of males with nonalcoholic steatohepatitis were non-obese (lean NAFLD) (33). To date, the susceptibility to develop lean NAFLD was higher among Asians (20%) as compared to Westerners, owing to body fat and muscle distribution (33, 34). Thus, it is clear that NAFLD is expected to be the biggest threat to hepatic-related poor health and mortality in South East Asia in future years. Therefore, healthy lifestyle interventions are required to be implemented in the general population to mitigate the heavy burden of smoking related NAFLD.

## Smoking and hepatocellular carcinoma

In recent years, Southeast and East Asia, reported an alarming rise in HCC cases according to Kim et al., with above 90% of primary liver cancers being diagnosed as HCCs (36, 37). Among Southeast Asian regions, Thailand reported a very high incidence of 22.3 per 100,000 in a year (38). In 2020, strikingly high percentages of HCC-related mortality around 545,202 and HCC incidence which was 590,952 were observed in the far east and Southeast Asia, especially in Cambodia, Thailand, and Mongolia, likely contributable to alcohol abuse, metabolic syndrome, and hepatitides B and C (3). Moreover, the rising trends were as follows: Vietnam (26,418), Thailand (27,394), Japan (45,663), and China (410,038) (3). In 2019, hepatocellular carcinoma (HCC) was the sixth most often diagnosed condition and the third leading cause of cancer deaths globally, according to the World Health Organization (39, 40). More than any other disease, HCC rates in the United States have tripled in the last 40 years and are expected to rise significantly in the next 20 years, unlike many other forms of cancer (40, 41). The causes include hepatitis B (HBV), hepatitis C (HCV), obesity, alcoholism, non-alcoholic fatty liver disease (NAFLD), cigarette use, hemochromatosis, aflatoxins in food, hereditary factors, and a variety of environmental carcinogens, such as arsenic and mercury (39–42). According to several studies in the United States, Europe, and Asia, smoking increases the chance of developing hepatocellular carcinoma (HCC) (42). Despite the fact that the hepatitis (especially HBV and HCV) and HCC are well-known, doctors may be reluctant to recognize the relationship between smoking and HCC. A Southeast Asian cohort study conducted among Chinese individuals assessed that individuals who never smoked were at a lower risk of developing HCC than those who smoked (43). Another study involving above 12 lac Korean individuals demonstrated a high-risk relationship of cigarette smoking with HCC related mortality in men (36). HCV/HCV-infected individuals with HCC who smoke are more likely to die than those who don't, according to a study in Bern, Switzerland (HR 2.99, 95 percent CI: 1.7–5.23, p 0.001) (42). 16 Smoking was directly responsible for 13% of all cases of hepatocellular cancer (41). Lee and colleagues found that the adjusted meta-RR for liver cancer in current smokers was 1.51 (95 percent confidence range 1.37–1.67) whereas it was 1.12 in former smokers, according to their research (95 percent confidence interval 0.78–1.60) (44). As many as 4,000 harmful components are included in cigarettes, many of which are poisonous, mutagenic, and carcinogenic (40). The harmful compounds increase oxidative stress and activate stellate cells, which accelerates fibrosis (44). Furthermore, smoking leads to a diminished oxygen-carrying capacity of the blood, promoted by detrimentally high carboxyhemoglobin concentrations. Subsequently, this stimulates erythropoietin production and increased hematocrit, secondary polycythemia. A high rate of RBC destruction ensues, leading to increased erythropoietin production and secondary intestinal iron absorption. Iron, after being phagocytized by macrophages, gets concentrated in liver cells, thereby facilitating the oxidative stress and hepatic injury (31, 44). Manifold oncogenic constituents, such as vinyl chloride, tar, nitrosamine, and hydrocarbons are present in cigarette smoke. Further, a significant presence of 4-aminobiphenyl has been associated with the increased risk of hepatocellular carcinoma (31). The formation of reactive carcinogens is mediated by the metabolization of 4-ABP and PAH (16). Further, the surge of proinflammatory cytokines such as TNFα, IL-33, and IL-1β and telomere shortening also contribute to tumor formation and progression (16). The tumor-suppressing gene p53 is blocked by N-nitrosodimethylamine, 4-aminobiphenyl, and cadmium, which induce liver fibrosis and cancer (40, 44). The anti-hepatocarcinogenic action of geranylgeranoic acid may be reduced by smoking, which inhibits hepatic monoamine oxidase B (44). Smoking cessation may be an effective method of preventing early death in people with HCC. Treatment options for hepatocellular carcinoma (HCC) include surgical excision, local radiofrequency ablation, transcatheter artery chemoembolization (TACE), radioembolization, and systemic targeted medicines like sorafenib (39).

## Non-alcoholic fatty liver disease associated hepatocellular carcinoma

Over two decades, from 1995 to 2015, the incidence of fatty liver in Shanghai, China raised a stunning percentage from 3.87 to 43.6% (3). HCC rates were 0.5% for NAFLD patients and 2.8% for NASH patients in long-term follow-up studies of non-alcoholic fatty liver disease (39). They've all been related to steatosis and its progression to necrosis and fibrous necrosis, as well as the development of liver cancer, including obesity and diabetes (39). A person's risk of NAFLD and/or cancer is increased by 1.5–4 times if their BMI exceeds 30 (39). The major cause of mortality in NAFLD is fibrosis (45). NAFLD-induced liver fibrosis (NAFLD-fibrosis) affects around 40% of morbidly obese persons (F1, range 13–97%) (45). Obese people with NAFLD-fibrosis have been shown to be at increased risk of smoking (45). There is a direct correlation between alcohol use and the advancement of fibrosis in people with related liver diseases (46). One of the most common indicators of diabetes-induced inflammation is the release of tumor necrosis factor (TNF), interleukin 6, and decreased production of adiponectin (IR) (39). Increased IGF-1 production is seen in IR patients with hyperinsulinemia, a growth factor that promotes cell proliferation while blocking apoptosis (39). Reactive oxygen species (ROS) and mitochondrial dysfunction are worsened when FFA levels rise (ROS) (39). Redox stress activates JNK1, which in turn blocks the p53 tumor suppressor gene and the nuclear respiratory factor 1 (Nrf1) 13 gene (39).

## Electronic cigarette exposure and NAFLD

Tobacco is not igniting when a user inhales the nicotine from an e-cigarette, which is also called an electronic nicotine delivery system (ENDS) (47). In recent years, e-cigarettes have become more popular, particularly among young people (16). A cross-sectional study conducted in Malaysia demonstrated that the highest number of users were young college or university students (39%), with peer pressure playing a major role among many (70%) (48). Another study conducted in Surabaya, Indonesia reported the growing trend of e-cigarettes, intending to quit cigarette smoking (36%) and just to try (24%) (49). Threatening products are seen as a waste of time and money in attempts to de-normalize smoking (47). The misconception that vaping is a healthier alternative to cigarette smoking is one factor. As in Ronald A. Fisher's period, a paucity of data has made e-cigarettes a contentious topic in public health circles (16, 47). E-cigarettes and other vaping products include a number of substances that may be detrimental to the health of users. Liver function has been shown to be negatively impacted by a variety of chemicals, including carbon monoxide, metals, nicotine, and nitrosamines, among others (16). E-cigarettes have been linked to liver damage in several ways, according to research done in the lab and animals (16). In comparison to control mice, rats fed a western diet (NASH model) and exposed to e-cigarettes had significantly higher levels of hepatic lipid accumulation and hepatocyte mortality (47). Oxidative stress and necrosis, as well as changes in cholesterol and fat metabolism and circadian clock networks in the liver, are all related to the adverse effects of electronic cigarettes on the development of steatosis (47). It does not rely on AMPK signaling, unlike smoking (47). More than 400 additional genes showed significant differences in expression between the NASH model mice and controls who had not used e-cigarettes, including those involved in lipid metabolism and cholesterol synthesis, according to studies conducted on hepatic RNA sequencing in these animals (47). We hypothesize that in the setting of NAFLD, e-cigarettes may cause liver dysfunction and changes in lipogenesis (47). Even more harm is likely to be done as a result of mitochondrial dysfunction and damage to the DNA itself (16). Finally, the flavoring compounds employed in e-cigarettes may induce hepatocyte harm in their own right, as previously stated (16). We know that endothelial dysfunction is an important step in the chain of events that leads to liver injury, fibrosis formation, and hemodynamic dysfunction (16). To put it another way, the endothelium damage caused by harmful chemicals in e-cigarettes might play a key role in the development of liver injury (16).

## Other complications associated with smoking and NAFLD

A relationship between CVD and NAFLD may exist because of the documented metabolic and cardiovascular risk factors associated with NAFLD, such as pro-inflammatory and atherogenic molecules (9, 16). Steatosis with smoking may have a synergistic effect on cardiovascular disease (CVD) (9). Transient ischemic attacks and strokes have been connected to carotid stenosis and carotid plaques, as well (9). In a cross-sectional investigation of the Chinese population, a link between NAFLD and carotid stenosis was found (carotid artery disease) (9). Another Southeast Asian Chinese study by Zheng et al. also observed a link between NAFLD and subclinical atherosclerosis, as shown by CIMT and ba-PWV measurements of carotid intima-media thickness (50). This is why a study was done to show that the AST/ALT ratio and ba-PWV are independent predictors of cardiovascular disease. Greater levels of ba-PWV are linked to AST/ALT ratios above 13.1 (51). Moreover, a northwestern Malaysian study evaluated that among 180 subjects with hypercholesterolemia, 12.2 and 16.7% individuals reported high levels of ALT and AST, respectively (52).

A serious risk associated with smoking is primary biliary cholangitis (PBC), although limited evidence exists (31). The proinflammatory and immunosuppressive effects of smoking, with an increase of Th1 cells in the portal tracts, may aggravate liver fibrosis in PBC patients, according to one study (16, 53). For every pack-year increase in smoking intensity, there was a 3.2-fold increase in the probability of advanced fibrosis (95 percent CI: 2.018–6.294) (53).

Cirrhosis, the severe liver damage caused by cigarette smoking, increases a person's vulnerability to several kinds of infections throughout the body (54). Innate and adaptive immune responses are disrupted, the complement system malfunctions and the number of white blood cells drops as a consequence of cirrhosis (54). It was shown that cirrhotic persons were 2.5 times more likely to have peri-implant infections compared to non-cirrhotic people. Smokers were also more vulnerable to and more likely to develop serious infections (54).

67.5% of patients with biopsy-proven NAFLD had depressive symptoms, which were associated with the severity of the illness based on histology, according to a study (55). Study results show that more than 15% of NAFLD patients suffer from depression (OR: 1.29, 95 CI: 1.02–1.64) (55). Consequently, it may be argued that depression and NAFLD are connected (55). Many neurotransmitter pathways are affected by smoking, including those that are linked to the development of depression (55). Doctors should only use screening scores for mood disorders in patients with depression when clinically necessary since this has an adverse effect on treatment response (55).

The ALT-defined phenotype of metabolic dysfunction-related liver disease has been used by researchers to establish a link between NAFLD (MDLD) and an elevated risk of malignancies other than the liver, such as breast, colon, liver, lung, and prostate (MDLD) (8). It is NAFLD that drives the link between obesity and cancer (8).

## Smoking and liver transplantation

Among Southeast Asian countries, India with amazing efforts of the National Organ and Tissue Transplant Organization, reported the development of 550 transplant centers and is the third most common country in terms of organ transplantation, with liver transplantation most common after kidneys (56). A cross-sectional study conducted in Thailand reported the use, safety, and efficacy of a newer procedure called living donor hepatectomy to treat end-stage liver disease (57). Tobacco use has been linked to mortality or the requirement for long-term treatment (LT) in patients with biopsy-proven NAFLD (HR 2.62; 95 percent CI 1.67–4.10) (16). Patients in the last stages of liver disease, particularly those with severe alcoholic liver disease, benefit greatly from liver transplantation (16, 46). Tobacco smoking has been linked to poor liver transplant results in several studies (15, 16). An increased risk of non-graft-related death is attributed to poor heart and lung function as well as infection susceptibility and immune system dysfunction (15, 16). The prevalence of active smoking among patients before and after liver transplantation is 52 and 44%, respectively (15). Among alcoholic liver disease liver transplant recipients, cardiovascular events and cancer are notably identified as the leading causes of death (15, 16). Smokers, on the other hand, had a 79 percent greater chance of dying than non-smokers (15). In order to reduce the risk of postoperative problems, quitting smoking 4 weeks before surgery is recommended (15). Patients who have not been able to quit smoking before LT should be offered smoking cessation programs that focus on preventing relapses in both alcohol and tobacco use while also improving overall health (15, 16). As a result, quitting smoking may reduce the chance of mortality and other unpleasant effects (15).

## Conclusion

Smoking, being overweight, and insulin resistance all contribute to the growing worldwide pandemic known as NAFLD, which in recent years, has shifted toward increased prevalence in Southeast Asia, which causes the liver to accumulate triglycerides and free fatty acids. Liver fibrosis is permanent and often indicates a dismal prognosis. Future physicians will gain knowledge and the chance to provide more focused care as a result of the declining cost of the highest quality and powerful genetic examination. The same is true for biochemical testing and the development of sophisticated imaging. There is mounting evidence that smoking contributes considerably to NAFLD and causes the side effects linked to liver transplantation. Therefore, it is important to urge these individuals to use cutting-edge treatment strategies for quitting and reducing their smoking, such as behavioral therapies and nicotine replacement therapy. Additionally, preventative measures including individual medical, physical, and nutritional counseling combined with education, heightened awareness among doctors and the public, and government initiatives to create an atmosphere more conducive to healthy lifestyle choices might have a hugely beneficial impact. Thus, there is a higher chance that treating the aforementioned modifiable risk factors may stop the development of NAFLD steatosis from fibrosis to cancer. As a result, this would be a crucial therapeutic target to lower the risk of CVDs. Thus, this research emphasizes smoking and NAFLD as major clinical issues that pose threats to world health with special emphasis on Southeast Asia. It is necessary to do further research to examine potential pathophysiological mechanisms behind the link between smoking and NAFLD.

## Author contributions

All authors have contributed equally to the review and have approved the final draft for publication.
